# Can provision of additional arthroscopic video material improve accuracy of assessment of temporomandibular joint disorders by dental Non-experts vs. MRI alone: An exploratory study in 3rd to 5th year German dental students

**DOI:** 10.1007/s10006-025-01369-9

**Published:** 2025-03-26

**Authors:** Yannick Rösner, Lennard-Luca Brüning, Andreas Neff

**Affiliations:** 1 Department of Oral and Craniomaxillofacial Surgery, Faculty of Medicine, UKGM GmbH, University Hospital Marburg, Philipps-University, 35043 Marburg, Germany; 2 Department of Anesthesiology, Intensive Care Medicine, and Pain Therapy, St. Vinzenz Hospital, Academic Teaching Hospital of the University of Cologne, 50733 Cologne, Germany

**Keywords:** Temporomandibular joint, Temporomandibular disorders, Arthroscopy, Magnetic resonance imaging

## Abstract

**Purpose:**

This exploratory study aimed to determine whether non-experts in the field of temporomandibular disorders (TMDs) are capable of correctly assessing various intra-articular TMDs based on magnetic resonance imaging (MRI), and whether supplementing corresponding arthroscopic imaging findings will enhance accuracy of their assessments.

**Methods:**

Non-experts for the purposes of this study were recruited from German dental students. After a focused instruction on TMDs, they completed two evaluation rounds to identify and assess selected pathologies of arthrogenic TMDs in patient cases. Initially, participants were provided with MRI images only; in a second round, additional arthroscopic video material was provided. Statistical analysis was performed to evaluate responses, and inter-rater reliability among non-experts was determined.

**Results:**

94 participants evaluated a total of 30 MRI scans of the temporomandibular joint (TMJ) obtained from 27 patients who had also undergone arthroscopy. Their assessment showed the relatively highest agreement with the correct diagnosis for disc perforations (68.2-71.9%) and when using both MRI and arthroscopy material. Synovitis showed the lowest agreement, and was more successfully detected based on arthroscopy (47.2%) alone. Overall, there was only slight to fair agreement among the study participants across diagnoses (Kappa 0.074–0.358). Non-experts showed significant inconsistencies in interpreting MRI and arthroscopic imaging, with only limited concordance with the actual diagnosis and an agreement rate of less than 71.9%.

**Conclusion:**

Dental students/Non-experts are unlikely to effectively interpret MRIs for the management of intra-articular TMDs based solely on their academic training. Enhanced curricular and postgraduate education in this area is therefore strongly recommended.

## Introduction

Temporomandibular disorders (TMDs) represent a highly heterogeneous group of disorders affecting the temporomandibular joint (TMJ), the associated musculature and related structures. With a prevalence of around 10–15% in the general adult population worldwide, TMDs are one of the most common causes of pain in the orofacial region [1–3]. Given the wide spectrum of conditions encompassed by the term TMD, imaging techniques play an increasingly important role alongside clinical diagnostics in differentiating between various pathologies, especially in arthrogenic TMDs. Since patients with TMD-related complaints will initially seek clinical evaluation and treatment from dental practitioners, the question arises if dentists in general are able to effectively utilize these techniques for diagnostic purposes.

As pointed out above, imaging techniques are particularly valuable for cases involving intra-articular pathologies, which include a broad spectrum of conditions. These range from disc dysfunctions (internal derangement) to degenerative osseous changes, such as osteoarthrosis, and inflammatory diseases, such as rheumatoid arthritis and juvenile idiopathic arthritis, to name the most common disorders [4].

For differentiating between potential intra-articular causes, magnetic resonance imaging (MRI) is considered the gold standard and is often used alongside clinical evaluation to identify potential TMJ-related causes [5].

MRI is best suited to investigate intra-articular causes of TMDs, as it effectively aids in assessing soft tissue abnormalities, such as perforations and/or displacements of the articular disc, as well as bony alterations, with superior diagnostic accuracy [6–8]. While MRI findings in TMDs should always be corroborated with clinical findings, MRI, when performed by trained specialists, effectively complements clinical diagnostics for assessing intra-articular changes of the TMJ [9].

In cases of refractory disease progression, surgical diagnostic methods are increasingly favored alongside imaging techniques [10]. Arthroscopy in this context offers the advantage of simultaneously allowing further diagnostic evaluation within the joint space, while also providing the opportunity for immediate therapeutic intervention, if necessary [11]. However, in a previously published study, our working group was able to demonstrate that interpreting arthroscopic findings in patients with arthrogenic TMDs was challenging for the students recruited as non-experts in TMDs in this context. Despite the availability of tools such as severity scores, these non-experts were unable to accurately assess lesions [12]. Therefore, we concluded that interpretation of arthroscopic findings will pose a challenge for general dental practitioners and should thus be reserved for experts in the field of TMD.

As a tertiary care center specializing in TMDs, our department frequently observes that a substantial proportion of patients presenting for advanced diagnostics and treatment have already undergone respective MRI examinations. This suggests that MRI diagnostics, unlike arthroscopies, frequently be initiated by dentists, including those who may not be experts in arthrogenic TMDs, often to support further therapeutic and diagnostic decisions, particularly in complex or unclear cases.

However, several studies from Germany have demonstrated a generally poor understanding of TMDs amongst both dental practitioners and dental students, and particularly the relevant diagnostic approaches are often marked by significant uncertainty [13, 14]. As a consequence, many patients are referred to specialized centers, sometimes without a definitive diagnosis [15].

Since dentists frequently refer patients for MRI diagnostics and make therapeutic decisions based on MRI findings, they should possess at least a basic level of competence in interpreting these images.

To investigate this competence, the study employed a cohort of dental students in the clinical phase of their studies (6th–9th semester) who had completed an intensive theoretical ring course on TMDs. As no further knowledge on this topic is provided within the curriculum until licensure, this preparation rendered the cohort comparable to trainees in oral and maxillofacial surgery (OMFS) or dental practitioners without postgraduate training in TMDs, thus serving as proxies for general dental practitioners.

The objective of this study was (a) to assess the current level of knowledge among German dental students in the clinical phase (6th–9th semester) as proxies for dental practitioners regarding the interpretation of MRIs in the clinical presentation of arthrogenic TMDs, and to determine whether they are capable to correctly evaluate MRIs in this context; and (b) to investigate whether the inclusion of images visualizing underlying pathologies (i.e., relevant arthroscopic imaging material) would enhance the accuracy of assessment of these conditions.

We hypothesized that non-experts in TMDs without extensive experience in this field would not achieve accurate diagnostic results based solely on MRIs, but we assumed that visualization with arthroscopic imaging material would improve the assessment of the respective pathologies. While no studies currently confirm that arthroscopic material improves diagnostic accuracy of clinical and /or MRI-based diagnostics, it seems plausible that supplying direct visualization of the underlying pathologies would optimize their identification.

## Materials and methods

### Imaging

Inclusion criteria were complete datasets for TMDs patients comprising MRI imaging, arthroscopic video material and information on the corresponding arthrogenic TMDs complaints. Exclusion criteria was incomplete data-set, TMD of non arthrogenic origin and arthroscopic imaging material already used in a previous study of our working group [12].

All patients whose arthroscopic imaging was used for the study underwent arthroscopy in the Department of Oral and Craniomaxillofacial Plastic Surgery at the University Hospital Giessen and Marburg between 2009 and 2022 due to TMD-associated complaints.

Among the patient cases included, the intraoperative arthroscopic video recordings (AV) were reviewed to identify representative pathologies associated with the underlying TMDs. The cases for the study were then randomly selected using a random number generator (Y.R.).

**Fig. 1:**
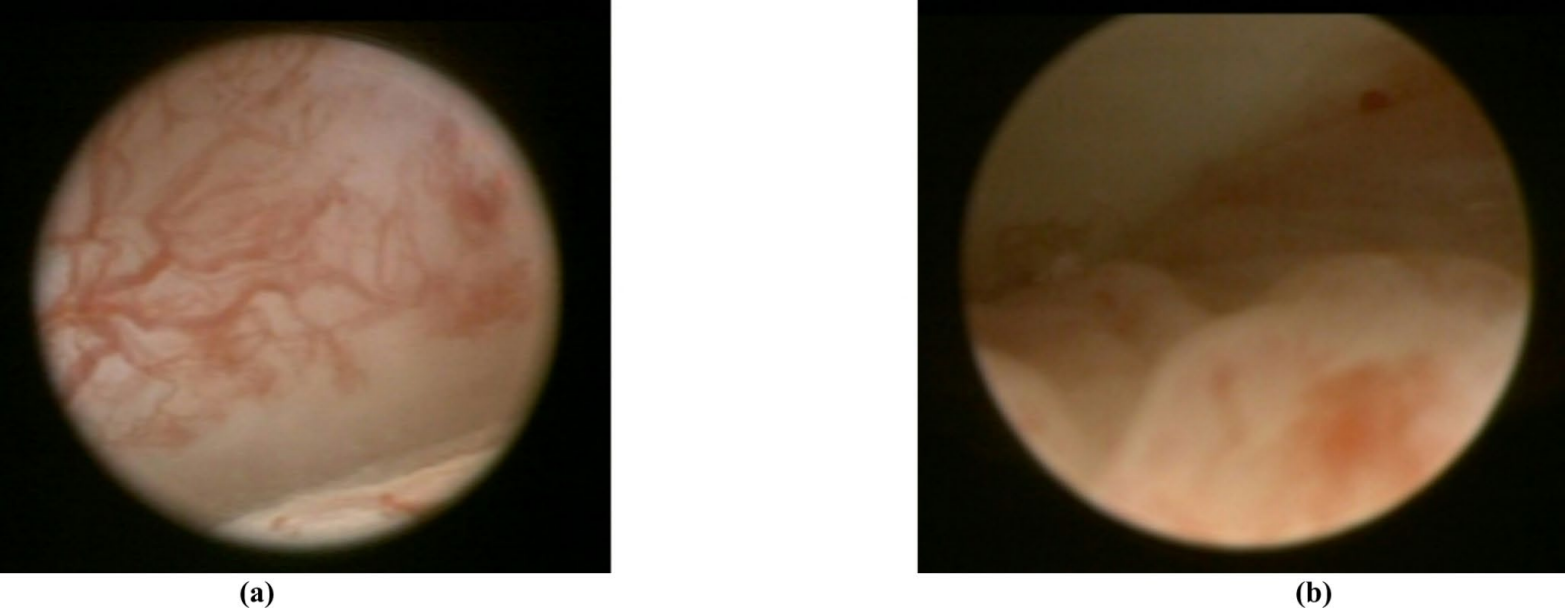
Screenshots taken from an arthroscopic video of the upper joint cavity. (**a**) showing a typical case of synovitis with vascular sprouts; (**b**) showing swelling and thickening of the posterior band, related to inflammation due to disk displacement with reduction (DDwR)

The intraoperative arthroscopic videos had been recorded routinely during arthroscopies under general anesthesia, and all patients had been operated on by the same surgeon (A.N) using Storz endoscopes (zero degree arthroscope, ∅ 4 mm; 30 degree arthroscope, ∅ 2.4 mm, KARL STORZ SE & Co. KG, Germany). The arthroscopic video recordings were then edited by the second and the senior author (L.B.; A.N.) into representative approximately 30-second clips, in which the pathologies of the corresponding patient were clearly visible (Fig. 1). In case of highly similar pathologies the case offering the best visualization was included to avoid redundancies.

**Fig. 2:**
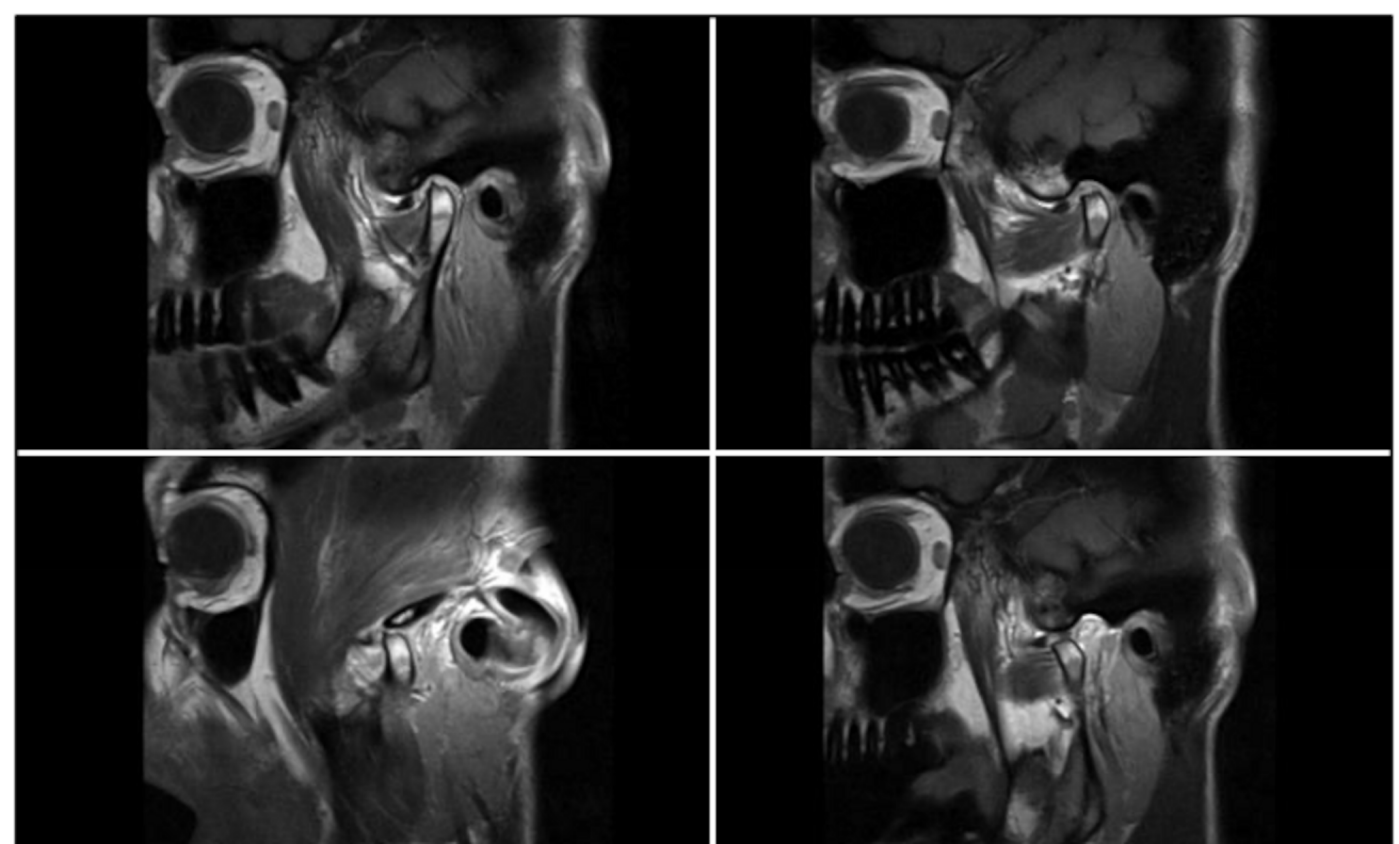
Representative sagittal T1-weighted MRI sections. The upper images display the TMJ in medial (right) and lateral (left) view respectively with the mouth closed. The lower images show the same medial (right) and lateral (left) views with the mouth open. Degenerative changes in the condylar head, as well as disc displacement are visible

The MRI images used were predominantly non-inhouse images from different radiological institutes, therefore produced on varying devices. Nevertheless, all recordings were taken in T1 or T2 weighting, and in all cases MRI recordings in closed- and open-mouth position were available. For the subsequent presentation of the MRI recordings, image series in sagittal and coronal sections were provided (Fig. 2).

The information on the clinical-radiological findings and postoperative diagnoses were obtained from the corresponding digitized patient records and transferred anonymously into a Microsoft Excel 2016 document (Microsoft 212 Corporation, Redmond, WA, USA). All postoperative diagnoses were made by the third author (A.N.), being an European-board-qualified academic oral and maxillofacial surgeon (EBOMFS) with long-standing experience in TMDs diagnostics and arthroscopy, holding a special qualification for functional diagnostics and therapy in TMDs accredited by the German Society of Functional Diagnostics and Therapy (DGFDT).

Each patient case was then categorized into at least one of five predetermined typical subcategories of intra-articular and degenerative TMDs as defined by AXIS I of the DC-TMD [16], based on the clinical-radiological diagnosis, established by specialized head and neck radiologists, and the postoperative diagnoses made following arthroscopy. These pathologies included disc displacement, disc perforation, fibrous adhesions, degenerative changes, and synovitis. The classification system was based on our previous study [12], with the modification of added subcategories for disc displacements and perforations. If the available patient cases could not be classified into any of these categories, they were excluded from the study. The same applied for cases in which rare pathologies, such as pigmented villonodular synovitis or synovial chondromatosis would have rendered a clear identification of the underlying pathology impossible.

The arthroscopic images used were also processed and evaluated accordingly. For further details with regard to preparation of the arthroscopic material cf. also [12]. Use of arthroscopic material in a previous study was an exclusion criterion.

### Study cohort

The study participants were recruited from the dental students attending the curricular lecture in OMFS at the Philipps University Marburg, Germany.

Inclusion criteria were: dental student in the clinical phase of their studies between the 6th and 9th semester, and student under the German dental licensing law valid at the time, i.e., the curriculum applicable for beginners until 2021.

Furthermore, participating students had to have passed the preclinical exam to be taken after the 5th semester at the time. After successfully passing this examination, basic scientific and anatomical knowledge will be present, and students are eligible to take part in clinical courses such as the curricular OMFS lectures.

In addition, participants had to have successfully participated in the curricular OMFS lectures, which, as module of their five-semester OMFS training, covers theoretical knowledge of TMDs, its pathologies, diagnostics and therapy, including approximately 50 lecture hours on TMDs and associated arthrogenic pathologies, over the semester. They were also required to have demonstrated command of diagnostic/therapeutic tools such as MRI and arthroscopy. Exclusion criteria for participating in the study were physical disabilities that would result in insufficient optical recognition of the MRI imaging material and/or the arthroscopic video material. All participants had to be at least 18 years of age. Additionally, they had to submit completed forms for both the first and second round of the study.

If all criteria were met, participants were required to give their informed consent for being included in the study before taking part. The study was conducted in accordance with the Declaration of Helsinki, and the protocol was approved by the Ethics Committee of the Faculty of Medicine at Philipps-University Marburg (IRB, project identification code 23-61BO) on 26.08.2023.

**Fig. 3:**
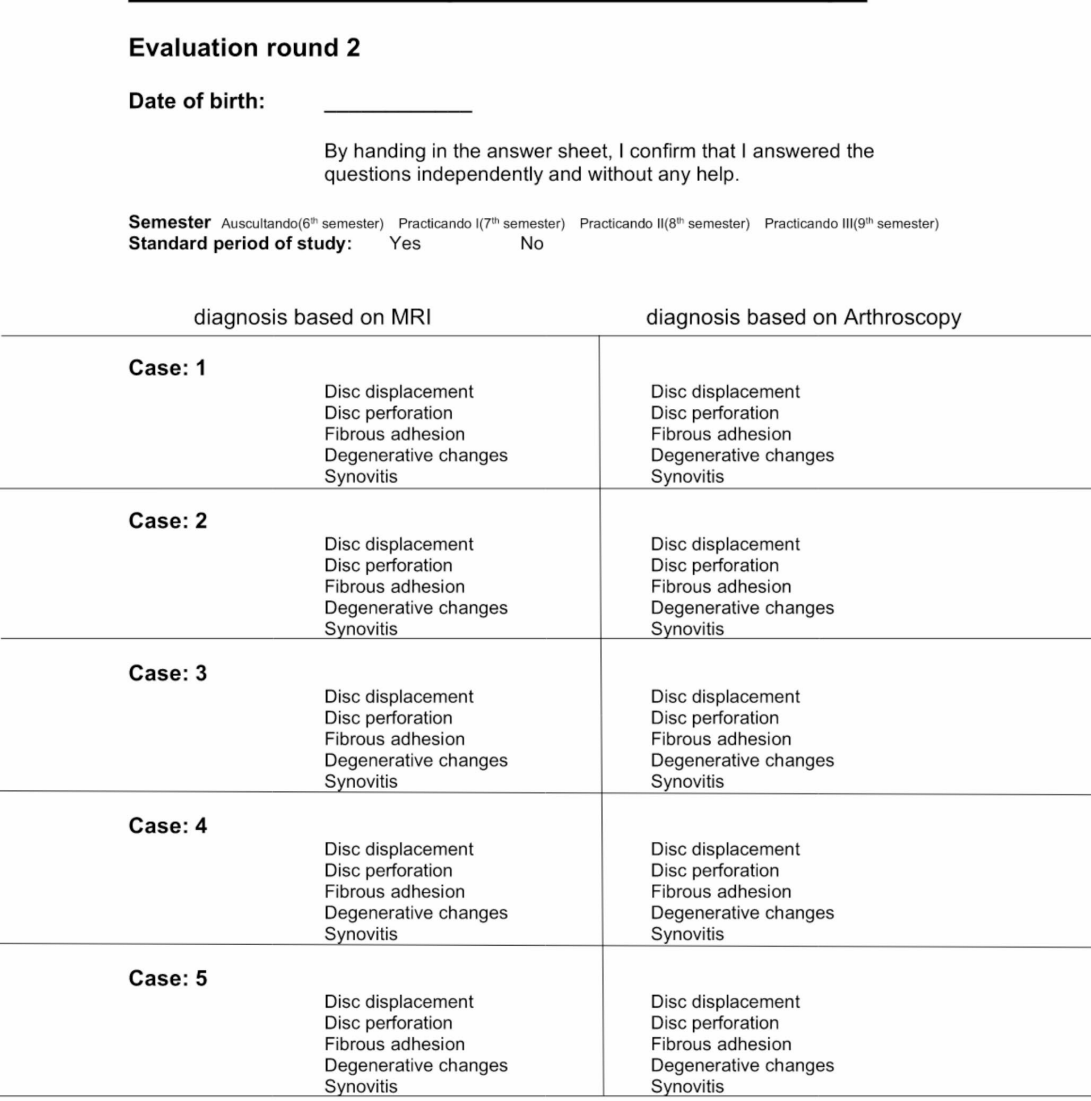
Evaluation form for the second round (T2), translated into English

This study was designed as a repeated cross-sectional study, to take place in two consecutive rounds after the end of the instructional lecture course on TMDs. In a first round (T1), each subject received MRI material of the patient cases, to be allocated by the subjects to at least one of the five pathologies mentioned above, using an evaluation form (Fig. 3). The participants were given exactly 90 s for each patient case.

In the subsequent second round (T2), conducted one week later, arthroscopic video material (AV) of the same patient cases was supplemented to the material used in the first round, however, the order of the patient cases was changed. In this round, the participants were given exactly 150 s per patient case (MRI 90 s; AV 60 s). The duration of the arthroscopic recordings was aligned to our previous study, which also had allowed 60 s for the evaluation of the arthroscopic material [12].

The patient cases were to be reassigned to the corresponding pathologies. In the first step, the evaluation was based solely on the MRI (MRI2). In the second step, after reviewing and utilizing both the arthroscopic video material and the MRI, the diagnoses were recorded in the corresponding column of the evaluation form (AV). The non-experts were able to modify or supplement their responses based on the additional information gained.

### Data collection and statistical analysis

After completion of the evaluations, the raw data from the study was transferred to a Microsoft Excel 2016 document by the first author (Y.R.).

An initial analysis of the sample size and corresponding effect size could not be determined in advance due to the unpredictable interdependencies in the data; such calculations would have been estimates, as a multilevel analysis was modeled with test subjects and patient cases as random factors. The sample size was considered adequate, as it was based on the results and pilot tests from our preliminary study, which utilized the same cohort size and a similar number of patient cases [12]. Furthermore, we were limited in cohort size, as no more than 100 students attended the corresponding OMFS course and were therefore eligible for the study.

The pathologies marked as correct by the subject in the respective case were labelled as 1, missing markings as 0. The acquired data sets were divided into three groups: MRI1 (T1), MRI2 (T2), and AV (T2). The evaluation of the second round was conducted separately for MRI and AV to allow for better differentiation of the results, particularly to enable a comparison between the assessments of MRI1 and MRI2, thereby ensuring that participants were not simply guessing.

These grouped data sets were then compared to the relevant postoperative clinical radiological diagnosis of the patient. The subjects’ responses were recoded as correct = 1 if their answer agreed with the diagnosis, and as incorrect = 0 if it did not.

The repeated measures were taken into account in a generalized linear mixed model: patient cases and subjects were modeled as random factors, diagnostic method and diagnosis as repeated-measure factors and fixed factors. The procedure GENLINMIXED (IBM SPSS Statistics for Windows, Version 26.0. Armonk, NY: IBM Corp) was used.

Since the dependent variable was binary (incorrect = 0; correct = 1), the model was computed as a logistic regression.

Since the hypotheses about the five diagnoses were considered to be independent of each other, no correction of p-values for multiple testing was performed (*p* < 0.05 = significant).

A Fleiss’ Kappa was then used for measuring the degree of interrater reliability. Kappa values were interpreted according to Landis and Koch [17]: ≤ 0 indicating poor agreement, 0.00–0.20 slight, 0.21–0.40 fair, 0.41– 0.60 moderate, 0.61–0.80 substantial, and 0.81–1.00 almost perfect agreement.

## Results

A total of 30 joints from 27 different patients were evaluated for the study (Table 1). The patient group consisted of 24 (89%) females and 3 (11%) males, with an average age of 45.89 years (± 13.52).

18 (60%) of the joints examined exhibited displaced discs, including both disc displacements with (DDwR) and without reduction (DDwoR). Inflammatory processes of the synovial membrane were observed intraoperatively in 21 (70%) of the patients. Disc perforations (5 cases, 17%), fibrous adhesions (6 cases, 20%) and degenerative changes (10 cases, 33%) were observed less frequently in the patient cohort.

94 students agreed to participate in the study and returned completed forms. The distribution of semesters was as follows: 24 students in their 6th semester (Auscultando), 21 students in their 7th semester (Practicando I), 23 students in their 8th semester (Practicando II) and 26 students in their 9th semester (Practicando III).

**Table 1:** Study population and participant cohort

	Number	Percentage
**Total number of patients**	27	100%
Male	3	11%
Female	24	89%
**Age ± Standard deviation**		45.89 ± 13.52
**Joints**	30	100%
Left	15	50%
Right	15	50%
**Postoperative diagnosis**		
Disc dislocation	18	60%
Disc perforation	5	17%
Fibrous adhesion	6	20%
Degenerative changes	10	33%
Synovitis	21	70%
**Total number of participants**	94	100%
6th semester	24	26%
7th semester	21	22%
8th semester	23	24%
9th semester	26	28%

Table 2 illustrates that, considering the fixed effects, the correlation between the diagnosis and the diagnostic method is highly significant. This indicates that the diagnosis varies significantly depending on the diagnostic method employed.

Given the scale of the sample size and the corresponding significance of potentially small correlations, the results were predominantly interpreted using estimated marginal means and contrast estimates, which represent the percentage of correct diagnoses made by the participants and highlight the differences across the three groups.

**Table 2:** Fixed effects

Source	F	df1	df2	*p*-value
**Corrected Model**	168.323	14	39,474	< 0.001
**Diagnosis**	506.625	4	39,495	< 0.001
**Diagnostic method**	17.345	2	39,465	< 0.001
Diagnosis * Diagnostic method	44.545	8	39,466	< 0.001

### Comparison of MRI and arthroscopy

Table 3 presents the differences between the accuracy of diagnoses as obtained from the diagnostic methods.

Disc displacements were accurately identified in 40.8–51.2% of cases (MRI1 51.2%; 95%, CI*: 47.8–54.6%; MRI2: 49.2%; 95%, CI: 45.8–52.7%; AV: 40.8%; 95%, CI: 37.6–44.2%).

For fibrous adhesions, the agreement with the postoperative diagnosis ranged from 53.8 to 70.4% (MRI1 70.4%; 95%, CI: 67.4–73.3%; MRI2: 66.5%; 95%, CI: 63.4–69.6%; AV: 53.8%; 95%, CI: 50.4–57.2%).

Regarding degenerative changes, all diagnostic methods achieved average accuracy in above 53.1% of assessments made (MRI1 59.3%; 95%, CI: 56–62.6%; MRI2: 53.1%; 95%, CI: 49.7–56.5%; AV: 55.9%; 95%, CI: 52.5–59.3%).

Regarding the evaluation of disc perforations, study participants demonstrated the highest level of accuracy across all three diagnoses made (MRI1 68.2%; 95%, CI: 65.1–71.1%; MRI2: 71.9%; 95%, CI: 69–74.7%; AV: 70.5%; 95%, CI: 67.5–73.4%).

For synovitis, MRI1 and MRI2 yielded the lowest agreement, with AV results showing relatively better agreement (MRI1 36.7%; 95%, CI: 33.5–39.9%; MRI2: 33.7%; 95%, CI: 30.7–36.9%; AV: 47.2%; 95%, CI: 43.8–50.6%). However, none of the results exceeded 50% agreement.

Table 4 presents a comparison of the various methods. In most cases, the differences observed in accuracy of diagnosis between the methods are statistically significant. Particularly noteworthy are the results for fibrous adhesions, where both MRI1 and MRI2 demonstrated outcomes superior to AV by over 10% (MRI1-AV: 16.6%; 95%, CI: 14–19.2%; MRI2-AV: 12.7%; 95%, CI: 10.1–15.3%).

Similar trends were observed for disc displacements, although the differences between MRI and AV results were less pronounced (MRI1-AV: 10.4%; 95%, CI: 7.7–12.9%; MRI2-AV: 8.4%; 95%, CI: 5.8–11%). The small difference of 1.9% between MRI1 and MRI2 was found to be statistically non-significant in this context.

For degenerative changes, MRI1 showed better results than both MRI2 and AV (MRI1-MRI2: 6.2%; 95%, CI: 3.6–8.8%; MRI1-AV: 3.4%; 95%, CI: 0.8–6%), while the AV value demonstrated slightly better agreement than MRI2 (MRI2-AV: 2.8%; 95%, CI: 0.2–5.4%).

Major differences were also noted in the detection of synovitis, where diagnoses based on/with AV outperformed both MRI1 and MRI2, resulting in a larger contrast between the methods (MRI1-AV: -10.6%; 95%CI: -13.2% to -8%; MRI2-AV: -13.5%; 95%CI: -16.1% to -10.9%).

Regarding the detection of disc perforations, there were no substantial differences between methods. Additionally, the differences observed in the comparison of MRI1 and MRI2 with AV (MRI1-AV: -2.3%; 95%, CI: -4.8% to -0.1%; MRI2-AV: 1.4%; 95%, CI: -3.8% to -1%) were found to be statistically insignificant (*p* > 0.05).

**Table 3:** Estimated marginal means of the different diagnoses

Disorder	Method	Percentage of correct diagnoses	Mean	Standard Error	95% CI*	
					lower	upper
**Disc displacement**	MRI1	51.2%	0.512	0.017	0.478	0.546
	MRI2	49.2%	0.492	0.017	0.458	0.527
	AV	40.8%	0.408	0.017	0.376	0.442
**Disc perforation**	MRI1	68.2%	0.682	0.015	0.651	0.711
	MRI2	71.9%	0.719	0.014	0.690	0.747
	AV	70.5%	0.705	0.015	0.675	0.734
**Fibrous adhesion**	MRI1	70.4%	0.704	0.015	0.674	0.733
	MRI2	66.5%	0.665	0.015	0.634	0.696
	AV	53.8%	0.538	0.017	0.504	0.572
**Degenerative changes**	MRI1	59.3%	0.593	0.017	0.560	0.626
	MRI2	53.1%	0.531	0.017	0.497	0.565
	AV	55.9%	0.559	0.017	0.525	0.593
**Synovitis**	MRI1	36.7%	0.367	0.016	0.335	0.399
	MRI2	33.7%	0.337	0.016	0.307	0.369
	AV	47.2%	0.472	0.017	0.438	0.506

*Confidence Intervals

**Table 4:** Pairwise comparison between the diagnostic tools

Diagnosis	Comparison	Percentage Difference	Contrast Estimate	Standard Error	t	df	*p*-value	95% CI	
								lower	upper
**Disc displacement**	MRI1 - MRI2	1.9%	0.019	0.013	1,427	39,462	0.154†	-0.007	0.046
	MRI1 - AV	10.4%	0.104	0.013	7.731	39,475	1.088e-14	0.077	0.129
	MRI2 - AV	8.4%	0.084	0.013	6.292	39,473	3.166e-10	0.058	0.110
**Disc perforation**	MRI1 - MRI2	-3.7%	-0.037	0.012	-3.035	38,566	0.002	-0.061	-0.013
	MRI1 - AV	-2.3%	-0.023	0.012	-1.887	39,430	0.059†	-0.048	0.001
	MRI2 - AV	1.4%	0.014	0.012	1.152	39,489	0.249†	-0.010	0.038
**Fibrous adhesion**	MRI1 - MRI2	3.9%	0.039	0.012	3.140	38,747	0.002	0.015	0.064
	MRI1 - AV	16.6%	0.166	0.013	12.729	17,123	0.000	0.140	0.192
	MRI2 - AV	12.7%	0.127	0.013	9.658	32,905	0.000	0.101	0.153
**Degenerative changes**	MRI1 - MRI2	6.2%	0.062	0.013	4.643	39,481	3.451e-06	0.036	0.088
	MRI1 - AV	3.4%	0.034	0.013	2.559	39,487	0.011	0.008	0.060
	MRI2 - AV	-2.8%	-0.028	0.013	-2.081	39,470	0.037	-0.054	-0.002
**Synovitis**	MRI1 - MRI2	3.0%	0.030	0.013	2.304	39,452	0.021	0.004	0.055
	MRI1 - AV	-10.6%	-0.106	0.013	-7.982	38,300	1.554e-15	-0.132	-0.080
	MRI2 - AV	-13.5%	-0.135	0.013	-10.278	32,987	0.000	-0.161	-0.109

†Adjusted to the lowest significant difference, the significance level is *p* > 0.05

### Agreement between the participants

Fleiss’ Kappa value was used to evaluate the agreement on diagnoses among the test subjects (Table 5). Overall, there was only a slight to fair agreement between the participants across all three diagnoses offered.

Degenerative changes exhibited the lowest agreement among all subcategories, with a Kappa value as low as 0.074 (95%, CI: 0.033 to 0.111). Similarly, for disc displacements, the agreement among study participants was also low, showing slight agreement (0.119; 95%, CI: 0.081 to 0.155). The results for fibrous adhesions (0.230; 95%, CI: 0.192 to 0.266), disc perforations (0.311; 95%, CI: 0.273 to 0.350), and synovitis (0.358; 95%, CI: 0.323 to 0.393) demonstrated fair agreement, with synovitis exhibiting the highest value.

**Table 5:** Kappa values across all diagnoses

Diagnosis	Kappa value	Agreement	Standard error	BCa 95% CI	
				lower	upper
**Disc displacement**	0.119	slight	0.019	0.081	0.155
**Disc perforation**	0.311	fair	0.019	0.273	0.350
**Fibrous adhesion**	0.230	fair	0.019	0.192	0.266
**Degenerative changes**	0.074	slight	0.019	0.033	0.111
**Synovitis**	0.358	fair	0.017	0.323	0.393

## Discussion

As Nebbe et al. and Orsini et al. have already pointed out for MRI diagnostics in general, the general diagnosis of TMDs is not always unequivocal and, more importantly, user-specific, even among specialized radiologists [18, 19]. Furthermore, differences in diagnoses based on arthroscopy results vs. MRI are evident, even among experts in blinded studies, when clinical information is missing [20].

As we demonstrated in our previous study, diagnosis and interpretation are particularly challenging in the specific area of TMD; we therefore aimed to establish whether non-experts might navigate MRI interpretation more effectively than interpretation of arthroscopic findings [12]. The aim of this study was to determine whether, and to what extent, non-experts in TMJ imaging are able to reliably assess subcategories of arthrogenic TMDs using MRIs, and whether complementing MRI with arthroscopic information is able to improve diagnostic accuracy.

Our findings did not reveal a clear pattern in this respect. Generally, it can be observed that, as expected, the results from MRI1 and MRI2 show less variability across most subcategories compared to the diagnoses based on or with the help of arthroscopic video material. Notable exceptions include disc perforations, where the results from all three methods are closely aligned (1.4-3.7%), as well as degenerative changes. The assessments made by the study participants varied less between MRI1 and MRI2 than between MRI and AV, which was anticipated, suggesting that MRI serves as a reliable baseline in these evaluations. As previously mentioned, the results were analyzed separately for better comparability. This clearly shows that the more similar assessments of MRI1 and MRI2 suggest that the non-experts did not guess their evaluations. The significant difference with AV highlights the effect of adding the arthroscopic video material. Regarding the agreement with the reference diagnosis provided by the primary examiner, relatively high concordance rates were observed for certain pathologies, such as disc perforations (68.2-71.9%) and fibrous adhesions (53.8-70.4%). However, for other pathologies, such as synovitis, more than half of the study subjects’ assessments did not align with the postoperative, arthroscopically verified diagnosis (36.7-47.2%), indicating a lack of familiarity with both arthroscopic and MRI findings.

When comparing the study participants’ results with those of other studies, usually conducted with experts, several significant differences emerge. These differences highlight the discrepancies between expert-based MRI assessments and the corresponding diagnoses compared to those made by less experienced subjects, a trend also observed in our previous study on arthroscopic pathologies [12].

Several studies have demonstrated that the degree of joint effusion and the inflammatory process correlate and are well visualized on MRI in cases of synovitis. Although the clinical-surgical, i.e., arthroscopic, method is generally considered to be more accurate and superior in this regard, the study subjects’ results in the study in hand were unexpectedly poor, regardless of the method used [21, 22]. Identifying joint effusion and synovitis on MRI and arthroscopic video material (Fig. 1) proved challenging (MRI1 36.7%; MRI2 33.7%; AV 47.2%). Nevertheless, it is evident that study participants were more confident in identifying synovitis using arthroscopic material (improvement of 10.6-13.5%) compared to MRI. This aligns with the findings of our previous study, where subjects had the least difficulty assessing this pathology accurately [12]. The overall poor performance, however, may be attributed to overinterpretation of the material provided.

In the assessment of disc perforations, the diagnostic instruments tested produced comparable results, which was contrary to our expectations. According to the literature, disc perforations can be reliably detected by experts using MRI, particularly when centrally located, although surgical intervention with visualization proved to produce even significantly superior results [8]. This should be especially true for smaller lateral perforations, such as those of the elongated laterodorsal ligaments, e.g., in cases of DDwoR. The study participants’ poor performance can likely be attributed to their lack of experience with arthroscopic findings, as pointed out above, even in typical pathologies. In this context it may be hypothesized that students and dental practitioners who usually do not undergo an extensive instructional lecture course on TMDs, such as the one offered to our dental students, in general may perform even worse.

Our study also found that MRI yielded better results when assessing disc displacements compared to AV (MRI1 51.2%; MRI2 49.2%; AV 40.8%), which was expected. Other authors have similarly demonstrated that disc displacements can be reliably and accurately detected using MRI techniques [6, 23, 24]. However, assessment based on arthroscopic video material was expected to produce similar results, which was not observed in this study cohort. The poorer performance could be due to the fact that only visual information recorded by the arthroscope was available to the participants, specifically, the tactile manipulations of the jaw by the surgeon, which are crucial for detecting potential joint movement restrictions (e.g. in cases of DDwoR vs. DDwR), were not visible. Consequently, it was evidently easier for the study subjects to visually assess disc position and the mobility of discs and condyles on MRI by comparing the respective closed and open mouth positions.

Comparing the results for degenerative changes, it is notable that both diagnostic methods yielded very similar outcomes (MRI1 59.3%; MRI2 53.1%; AV 55.9%). Depending on the severity of the changes, these should be clearly visible on MRI, even though bony changes can be better detected by CT or cone beam computed tomography (CBCT) [7]. Nevertheless, studies also show that accurate assessment of lesions is possible and reliable with arthroscopy [25].

In the results for fibrous adhesions, MRI assessments showed better outcomes compared to arthroscopy (MRI1 70.4%, MRI2 66.5%, AV 53.8%), which was contrary to our expectations. This once more raises the question of how closely the assessments by students or non-experts will reflect those done by experts. Some studies, such as by Zhang et al., demonstrate poorer results in the assessment of fibrous adhesions using MRI compared to arthroscopy [26]. The reason for this may again be misinterpretation of lesions due to lack of experience. The study participants appeared unable to distinguish between slight fibrous bands and pseudo-wall-like structures, potentially perceiving both as adhesions. However, our previous study also showed that the inclusion of lesion severity scores, such as those by Segami et al. [27], has little or no impact on non-expert evaluations [12].

When considering interrater reliability, it is evident that the study subjects did not consistently agree on the individual diagnoses, i.e., there was a higher degree of variation in the results, with only slight to fair agreement between participants (Kappa 0.074–0.358). This is consistent with our findings that students/non-experts, due to their lack of experience, face considerable difficulty in evaluating and recognizing various pathologies. This was also evident in their assessment of arthroscopic images alone [12].

Our study presents several limitations that may have affected the results. Firstly, the study cohort consisted exclusively of students from a single German university. While the participants demonstrated a relatively advanced level of knowledge having attended an OMFS course on TMDs [12], differing curricula in other dental schools with varying emphasis on TMDs and their diagnostics could possibly yield divergent results. Given that our cohort had recently been exposed to the topic of TMDs as part of the focused one-semester curricular course, as well as having been trained by our prosthodontic colleagues and specialists for TMDs accredited by the German Society of Functional Diagnostics and Therapy (DGFDT), it can be reasonably assumed that other subjects with less targeted preparation may have performed worse.

Through this intensive training—which represents the only TMD education provided during dental studies until licensure—our cohort has attained a level of proficiency comparable to, or perhaps even beyond, that of average practicing dentists, therefore rendering them suitable subjects for assessing TMD knowledge in (future) dental practitioners. This however, needs to be confirmed in further studies.

In this context, various studies suggest that the integration of modern teaching methods, both for MRI and temporomandibular joint arthroscopy, is able to enhance the understanding of TMDs and may potentially lead to overall better results [28, 29].

Recent studies also indicate that, alongside improvements in teaching methods for students, advancements in MRI diagnostic technology for TMDs have also been achieved. For example, the use of enhancement filters can improve the efficiency of assessments [30]. However, for our study, the images were sourced from different radiological institutes, resulting in the use of a variety of technologies.

Moreover, the choice of a repeated cross-sectional design may have been a limiting factor. The study subjects were allowed only limited time to evaluate each patient case. In clinical practice, radiologists have the opportunity to assess MRI scans for longer, and to repeatedly review the images if uncertainties arise. Our study participants were limited to 90 s for each assessment. A similar limitation applied to the arthroscopic images. Surgeons, during operations, will be able to assess joint lesions from multiple angles. Although the study subjects were provided with high-quality video material of the pathologies, the footage was only replayed once, and the position of the arthroscope was predetermined. Furthermore, as previously mentioned, the participants had no opportunity to replicate the jaw manipulations performed by the surgeon, which can also be crucial for accurate diagnosis.

As demonstrated in our previous study [12], our current findings again suggest that particularly the diagnosis of arthrogenic TMDs poses a significant challenge to non-experts and, therefore, should primarily be reserved for specialists in the field of TMDs. Furthermore, this study also revealed that among non-experts there was no substantial concordance with the accurate diagnosis overall, regardless of whether MRI and/or arthroscopy was used (< 71.9%). As previously mentioned, this aligns with the findings of other studies, which have also identified deficits and uncertainties in the general knowledge and management of TMDs in dentists [13, 14]. Another study, which examined the evaluation of TMDs by students, similarly reported low agreement when the assessment was based solely on MRI [31]. Nevertheless, some studies also highlight possibilities for improving the use of diagnostic tools. For example, online training programs have been shown to enhance MRI interpretation in the assessment of the TMJ [32]. Studies from other medical disciplines suggest that newer training methods, such as simulators for minimally invasive procedures, along with more intensive engagement with the subject, can contribute to long-term knowledge retention [33]. Repetition and reinforcement of acquired knowledge appear to be beneficial in this context.

Our results provide evidence to reject the null hypothesis (H0). We hypothesized that non-experts would achieve better results when pathologies were visualized, using arthroscopic video material, compared to MRI alone, and that MRI itself would not yield accurate diagnostic results. Although no studies currently demonstrate the benefit of arthroscopic video material in assessments by non-experts, it seemed plausible that direct visualization would improve diagnostic performance. However, this was not the case. While the results obtained using MRI were indeed not accurate, such visualization of pathologies did not improve the accuracy of assessment. As previously mentioned, the mere detection of disc displacements on MRIs, for example, appears to be easier for beginners, as is the case with pronounced adhesions. In our non-expert cohort, arthroscopy rarely outperformed MRI, and no significant improvement was achieved through the use of arthroscopy alone, except in the case of joint inflammation, which the non-expert participants were able to detect significantly better with the aid of arthroscopic imaging, despite generally lower confidence in their interpretation.

Overall, the results from our study suggest that the know-how provided so far during dental training in MRI diagnostics appears insufficient, i.e. it does not adequately prepare dentists-to-be, or currently practicing dentists, to accurately read MRIs, particularly in the context of arthrogenic TMDs. Our study cohort’s ability to accurately assess the underlying pathologies was limited in spite of intensive training on TMDs, and there was also a notable lack of agreement among them.

Future research on this topic could investigate, whether participation in extracurricular or postgraduate education programs in MRI diagnostics for arthrogenic TMDs leads to improved diagnostic outcomes. Furthermore, consideration should be given to modifying the study design to overcome current limitations, for example, by extending the evaluation time for imaging materials to minimize potential sources of error or by implementing clearer evaluation criteria to avoid overinterpretation. Lastly, a future comparison between general dentists and OMFS in MRI assessment would also be a valuable area of investigation.

## Conclusion

Our study indicates that the interpretation of MRIs for conditions within the spectrum of arthrogenic TMDs was challenging for the non-experts of our study cohort, consisting of dentists-to-be as proxies for future practicing dentists. No substantial concordance with the underlying pathologies tested for was observed. Even with the inclusion of arthroscopic imaging material, which provided added visualization of the pathologies, diagnostic accuracy remained low.

These findings suggest that dental students, and consequently also postgraduate dental practitioners, may not be able to proficiently interpret MRIs based solely on their academic training.

However, given that a basic understanding of correct handling and interpretation of MRIs is likely to benefit young professionals in dentistry and oral and maxillofacial surgery throughout their careers, more thorough training for this diagnostic competency is to be recommended, to be incorporated into the curriculum of dental schools and postgraduate training.

## Data Availability

The data presented in this study are available on request from the corresponding author.
